# Impact on in-hospital mortality of ceftaroline versus standard of care in community-acquired pneumonia: a propensity-matched analysis

**DOI:** 10.1007/s10096-021-04378-0

**Published:** 2021-11-12

**Authors:** Catia Cilloniz, Raúl Mendez, Héctor Peroni, Carolina Garcia-Vidal, Verónica Rico, Albert Gabarrus, Rosario Menéndez, Antoni Torres, Alex Soriano

**Affiliations:** 1 Department of Pneumology, Hospital Clinic of Barcelona, August Pi I Sunyer Biomedical Research Institute - IDIBAPS, University of Barcelona, Biomedical Research Networking Centers in Respiratory Diseases (CIBERES), Barcelona, Spain; 2 Department of Pneumology, Hospital La Fe de Valencia, Valencia, Spain; 3 Internal Medicine Department, Respiratory Medicine Unit and Emergency Department, Hospital Italiano de Buenos Aires, Buenos Aires, Argentina; 4 Department of Infectious Diseases, Hospital Clinic of Barcelona, C/Villarroel 170, 08036 Barcelona, Spain

**Keywords:** Community-acquired pneumonia, Severe pneumonia, Ceftaroline, Pneumonia, Antimicrobials

## Abstract

The purpose of this study is to evaluate the in-hospital mortality of community-acquired pneumonia (CAP) treated with ceftaroline in comparison with standard therapy. This was a retrospective observational study in two centers. Hospitalized patients with CAP were grouped according to the empiric regimen (ceftaroline versus standard therapy) and analyzed using a propensity score matching (PSM) method to reduce confounding factors. Out of the 6981 patients enrolled, 5640 met the inclusion criteria, and 89 of these received ceftaroline. After PSM, 78 patients were considered in the ceftaroline group (cases) and 78 in the standard group (controls). Ceftaroline was mainly prescribed in cases with severe pneumonia (67% vs. 56%, *p* = 0.215) with high suspicion of *Staphylococcus aureus* infection (9% vs. 0%, *p* = 0.026). Cases had a longer length of hospital stay (13 days vs. 10 days, *p* = 0.007), while an increased risk of in-hospital mortality was observed in the control group compared to the case group (13% vs. 21%, HR 0.41; 95% CI 0.18 to 0.62, *p* = 0.003). The empiric use of ceftaroline in hospitalized patients with severe CAP was associated with a decreased risk of in-hospital mortality.

**Supplementary Information:**

The online version contains supplementary material available at 10.1007/s10096-021-04378-0.

## Introduction

Approximately 10 to 18% of hospitalized patients with community-acquired pneumonia (CAP) present with severe pneumonia that requires admission to the intensive care unit (ICU) [1]. Severe CAP is associated with high mortality, ranging from 25 to more than 50% [2, 3]. Prompt identification of severe pneumonia and early, adequate antibiotic therapy are crucial in managing these cases. Based on this observation, early, adequate antibiotic therapy could reduce mortality in severe CAP.

Several studies have reported the beneficial effects on the patient outcomes by using ceftriaxone plus macrolide regimen as empiric therapy [4, 5]. However, this combination is not good enough to cover *Staphylococcus aureus*, which is increasingly identified as a cause of severe pneumonia with high associated mortality, particularly in cases related to influenza virus co-infection or COVID-19 [1, 6, 7]. Also, the increasing prevalence of methicillin-resistant *S. aureus* and penicillin- and ceftriaxone-resistant *Streptococcus pneumoniae* in severe CAP has made this combination of antibiotics less effective [8, 9]. Ceftaroline is a broad-spectrum cephalosporin that covers gram-positive bacteria (including methicillin-susceptible and methicillin-resistant *S. aureus* and drug-resistant *S. pneumoniae*) and third-generation-susceptible gram-negative bacilli [10]. Results of the FOCUS 1 and 2 trials demonstrated the superiority of ceftaroline against ceftriaxone in bacterial CAP; however, these studies did not include severe cases and the mortality rate was low [10]. In comparison with ceftriaxone, ceftaroline has shown to have superior in vitro activity against *S. aureus* (≥ 16-fold more potent than ceftriaxone against MSSA and active against MRSA), *S. pneumoniae*, and other common CAP pathogens [11, 12].

Using the propensity score matching (PSM) method, we aim to evaluate in-hospital mortality of CAP treated with ceftaroline in comparison with standard therapy (Supplementary Table 1) in our cohort of severe CAP.

## Methods

### Study design

This was a retrospective analysis of prospectively collected data conducted at two Spanish hospitals (Hospital 1 from 1996 to 2020, and Hospital 2 from 2017 to 2020). The collection method was systematic, and all patients with CAP admitted to both hospitals were enrolled in the study.

### Selection of patients

We enrolled all consecutive adult patients with a diagnosis of CAP admitted to hospital via the emergency department. We included patients from nursing homes, as we previously demonstrated that the microbial aetiology in this population is similar to that of CAP arising in people living in their own homes [13].

Exclusion criteria were as follows: (a) severe immunosuppression (AIDS, chemotherapy, immunosuppressive drugs [e.g., oral corticosteroid ≥ 10 mg prednisone or equivalent per day for at least 2 weeks]); (b) active tuberculosis; (c) cases with a confirmed alternative diagnosis.

The study was approved by the Ethics Committees of both institutions (Register: 2009/5451). The need for written informed consent was waived due to the non-interventional design.

### Definitions

CAP was defined as a new pulmonary infiltrate on chest x-ray performed at hospital admission, combined with symptoms and signs consistent with a lower respiratory tract infection (e.g., fever, cough, sputum production, and pleuritic chest pain) in patients with no recent hospitalization or regular exposure to a healthcare system. Severe CAP was diagnosed by the presence of at least one major or three minor criteria, as set out by the Infectious Diseases Society of America/American Thoracic Society (IDSA/ATS) guideline [14]. Prior antibiotic treatment was defined as antibiotics taken within the week before disease presentation. Polymicrobial pneumonia was defined as pneumonia due to more than one pathogen.

### Data collection

Demographic variables, comorbidities, and physiologic parameters were collected in the emergency department within 24 h of admission. Comorbidities of interest included chronic respiratory disease (e.g., chronic obstructive pulmonary disease, asthma, and bronchiectasis), diabetes mellitus, chronic cardiovascular disease, neurologic disease (e.g., dementia, coma, stroke, degenerative diseases, Parkinson’s disease, and Down syndrome), chronic renal disease, chronic liver disease, and previous neoplasm.

The Pneumonia Severity Index (PSI), CURB-65 score (i.e., confusion, urea nitrogen, respiratory rate, blood pressure, and age ≥ 65 years), and SOFA score were calculated at admission. During hospitalization, we recorded whether the patients had specific complications, including multilobar infiltration, pleural effusions, acute respiratory distress syndrome (ARDS), septic shock, or acute renal failure. Further details are reported elsewhere [15]. All surviving patients were visited or contacted by telephone 30 days after discharge.

### Microbiologic evaluation

Microbiologic examination was performed on respiratory, urinary, and blood samples. Cultures were collected before the initiation of empiric antibiotic therapy in the emergency department. Criteria for making an aetiologic diagnosis have been reported previously[15].

Blood cultures, sputum cultures, and urine samples for *S. pneumoniae* and *Legionella pneumophila* antigen detection were obtained within 24 h of hospital admission. When available, pleural fluid, tracheobronchial aspirates, and bronchoalveolar lavage samples were collected for Gram and Ziehl–Neelsen staining and processed for bacterial, fungal, and mycobacterial pathogen detection. The results of susceptibility testing were interpreted according to EUCAST guidance (http://www.eucast.org). Blood samples for serology of atypical pathogens and respiratory virus were collected at admission and between the third and sixth week thereafter.

Respiratory viruses were diagnosed by serology, immunofluorescence assay (IFA), and isolation in cell cultures between 2005 and 2007. However, between 2008 and 2019, polymerase chain reaction (PCR) and/or cultures of nasopharyngeal swab samples were used instead. Two independent nested multiplex real-time PCR tests were performed to detect human influenza viruses (A, B, and C), respiratory syncytial virus, adenoviruses, parainfluenza viruses (1–4), coronaviruses (229E and OC43), enteroviruses, and rhinoviruses (A, B, and C).

### Outcomes

Primary outcomes were in-hospital and 30-day mortality. Secondary outcomes included length of hospital stay, ICU mortality, length of stay in ICU, need of mechanical ventilation, and 30-day and 1-year mortality.

### Statistical analysis

We report the number and percentage of patients for categorical variables, the median (first quartile; third quartile) for continuous variables with non-normal distributions, and the mean (standard deviation) for continuous variables with normal distributions. Categorical variables were compared using the chi-squared test or Fisher’s exact test, whereas continuous variables were compared using the *t* test or nonparametric Mann–Whitney *U* test.

Patients receiving ceftaroline in monotherapy or in combination as empirical therapy were considered the case group, whereas the remaining cohort was considered as controls. PSM was used to obtain a balance between patients in the case and control groups. To match the two cohorts, we used a 1:1 nearest neighbor matching without replacement within a match tolerance width of 0.005. Variables were chosen for inclusion in the PSM calculation according to methods set forth by Brookhart et al. [16]. Variables included were associated with the case group and outcomes (age, gender, chronic respiratory disease, chronic cardiovascular disease, diabetes mellitus, neurologic disease, chronic renal disease, chronic liver disease, previous neoplasm, fever, confusion, C-reactive protein, PaO_2_/FiO_2_, polymicrobial, bacteremia, multilobar, septic shock, ICU admission, and mechanical ventilation). An adequate model fit with discrimination and calibration of the PSM was demonstrated by the logistic model including covariates yielded a goodness-of-fit *p* = 0.867.

Cox proportional hazard regression models were used for in-hospital, 3-day, and 30-day mortality. The hazard ratio (HR) and its 95% confidence intervals (CI) were calculated.

We used the multiple imputation method for missing data in the covariates of the PSM model.

The level of significance was set at 0.05 (two-tailed). All statistical analyses were performed using IBM SPSS Statistics 26.0 (Armonk, NY, USA).

## Results

### General clinical characteristics

**Fig. 1:**
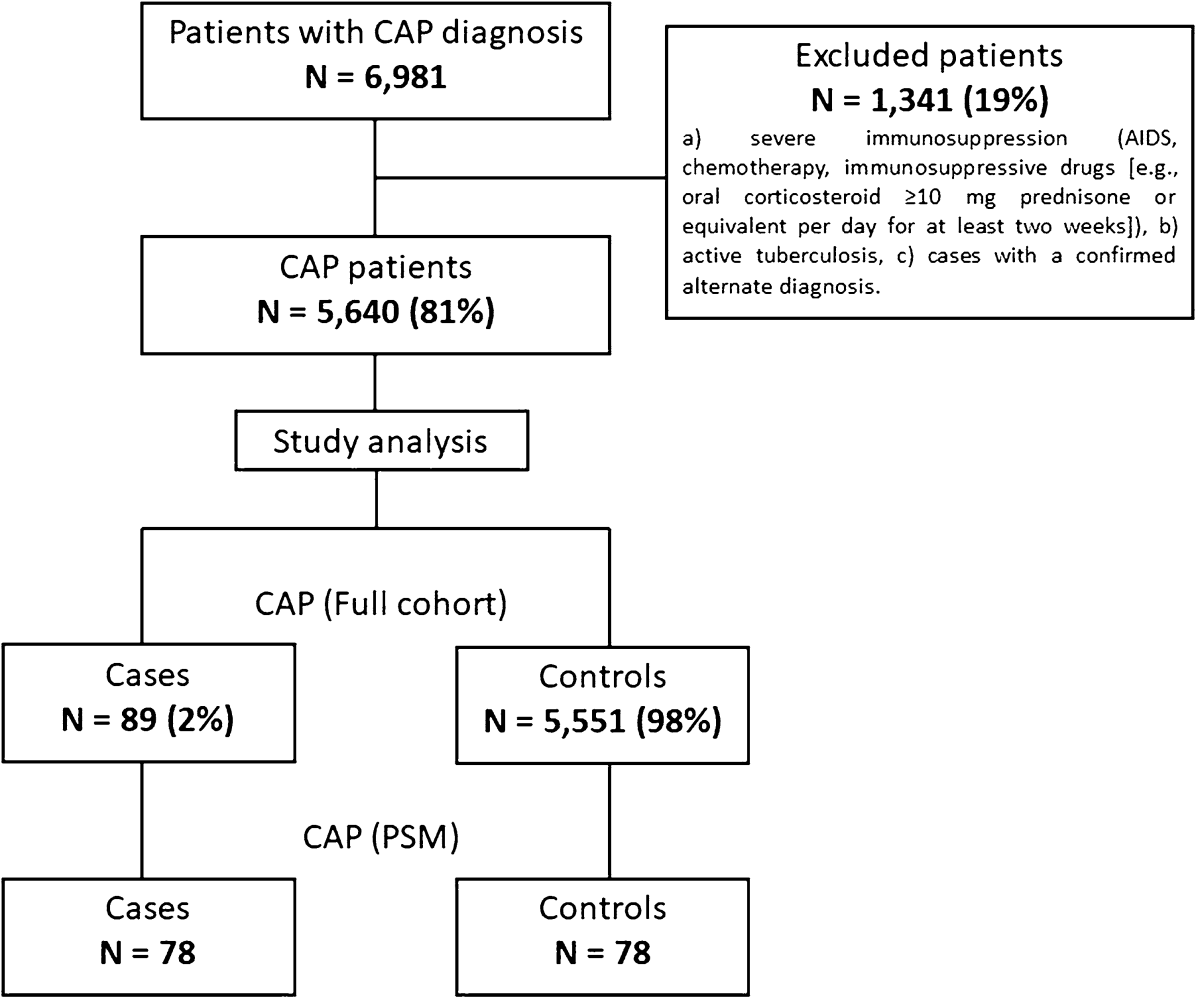
Flow diagram of study population

During the study period, 6981 patients with CAP were enrolled. Of these, 5640 (80%) patients met the inclusion criteria. In the full cohort, a total of 5551 (99%) received standard empiric antibiotic treatment and 89 (1%) received ceftaroline (3% monotherapy and 97% in combination therapy [46% ceftaroline + azithromycin; 16% ceftaroline + levofloxacin; and 38% other combinations]). After PSM was performed, 156 patients were finally included in the study (78 cases and 78 controls) (Fig. 1).

Table 1 summarizes the demographics and clinical characteristics of patients between case and control groups in the full cohort and after PSM. In the full cohort, and when compared to the control group, the case group was more likely to be younger and have a lower rate of influenza vaccines and a higher rate of pneumococcal vaccine. Also, the case group was less likely to have previously received inhaled corticosteroids and more frequently had chronic renal disease and a previous neoplasm. At admission, the case group less frequently presented with fever and purulent sputum than the control group. At admission, the case group had higher levels of C-reactive protein, lower lymphocyte counts, and worse oxygenation. Also, the case group at days 2 and 3 had higher levels of C-reactive protein and neutrophils, yet a lower rate of lymphocyte counts. In comparison with the control group, the case group also had a higher percentage of patients with severe CAP, with more cases of complications including bacteremia, multilobar involvement, ARDS, and acute renal failure. Pneumococcal vaccination, purulent sputum, and pleuritic pain were the only general variables not adequately balanced between both groups after PSM.

**Table 1:** Patient characteristics and outcomes

	CAP (full cohort)			CAP (propensity score matching)		
Variable	(*N* = 5640)			(*N* = 156)		
	Case (*n* = 89)	Control (*n* = 5551)	*p* value	Case (*n* = 78)	Control (*n* = 78)	*p* value
Age, mean (SD), years	64 (17)	70 (17)	**0.001**	64 (17)	65 (19)	0.673
Male sex, *n* (%)	52 (58)	3,456 (62)	0.460	48 (62)	50 (64)	0.740
Current smoker, *n* (%)	18 (31)	1,223 (22)	0.129	18 (31)	20 (26)	0.518
Current alcohol use, *n* (%)	4 (7)	787 (14)	0.097	4 (7)	10 (13)	0.240
Previous antibiotic, *n* (%)	16 (19)	1,222 (23)	0.391	15 (20)	17 (23)	0.690
Influenza vaccine, *n* (%)	23 (29)	1,833 (45)	**0.004**	20 (29)	19 (35)	0.543
Pneumococcal vaccine, *n* (%)	26 (32)	822 (20)	**0.010**	24 (34)	10 (18)	**0.045**
Previous inhaled corticosteroids, *n* (%)	9 (10)	1,053 (19)	**0.034**	7 (9)	15 (19)	0.065
Previous systemic corticosteroids, *n* (%)	3 (3)	217 (5)	0.525	3 (4)	4 (6)	0.701
Previous episode of pneumonia, *n* (%)	16 (18)	745 (14)	0.291	15 (19)	9 (12)	0.232
Comorbidities, *n* (%) ^a^	55 (62)	4,074 (74)	**0.013**	48 (62)	55 (71)	0.237
Chronic respiratory disease	32 (36)	2,472 (45)	0.080	29 (37)	32 (41)	0.623
Chronic cardiovascular disease	10 (11)	918 (17)	0.176	9 (12)	14 (18)	0.259
Diabetes mellitus	16 (18)	1,178 (21)	0.433	13 (17)	13 (17)	> 0.999
Neurologic disease	10 (11)	1,007 (19)	0.079	9 (12)	13 (17)	0.357
Chronic renal disease	13 (15)	473 (9)	**0.044**	11 (14)	14 (18)	0.513
Chronic liver disease	5 (6)	272 (5)	0.627	5 (6)	3 (4)	0.719
Previous neoplasm	16 (18)	512 (10)	**0.008**	14 (18)	15 (19)	0.837
Nursing home, *n* (%)	2 (3)	364 (7)	0.434	2 (3)	4 (5)	0.696
Cough, *n* (%)	68 (77)	4,270 (78)	0.882	58 (75)	60 (7)	0.815
Purulent sputum, *n* (%)	40 (45)	3,097 (57)	**0.024**	32 (42)	51 (68)	**0.001**
Dyspnea, *n* (%)	62 (70)	3,907 (72)	0.818	54 (70)	59 (78)	0.291
Pleuritic pain, *n* (%)	23 (26)	1,889 (35)	0.109	18 (24)	34 (44)	0.009
Fever, *n* (%)	56 (63)	4,157 (76)	**0.006**	46 (59)	48 (62)	0.744
Confusion, *n* (%)	12 (13)	1,147 (21)	0.090	11 (14)	10 (13)	0.815
C-reactive protein at baseline, median (IQR), mg/dL	25.3 (13.9; 39.4)	17.8 (8.5; 27.3)	** < 0.001**	22.6 (10.3; 31.6)	25.1 (16.4; 33.9)	0.244
C-reactive protein at days 2 and 3, median (IQR), mg/dL	25.3 (15.5; 31)	15.3 (7.4; 24.1)	** < 0.001**	24.5 (15.5; 30.6)	20.6 (8.5; 28)	0.269
Neutrophils at baseline, median (IQR), cell/mm^3^	9,810 (4,686; 15,032)	10,160 (6,622; 14,661)	0.380	9,626 (5,376; 14,711)	10,640 (7,304; 14,280)	0.388
Neutrophils at days 2 and 3, median (IQR), cell/mm^3^	11,550 (7,650; 17,606)	8,306 (5,465; 12,466)	**0.009**	11,659 (7,769; 17,606)	8,835 (5,192; 12,220)	0.081
Lymphocytes at baseline, median (IQR), cell/mm^3^	660 (380; 1192)	900 (543; 1,386)	**0.001**	661 (386; 1,152)	900 (428; 1,463)	0.135
Lymphocytes at days 2 and 3, median (IQR), cell/mm^3^	600 (387; 816)	902 (531; 1,379)	**0.003**	592 (387; 753)	722 (520; 1,122)	0.057
PaO_2_/FiO_2_, median (IQR)	238 (173; 295)	276 (233; 316)	** < 0.001**	241 (177; 300)	248 (184; 310)	0.656
PSI score, median (IQR)	104 (76; 135)	101 (78; 126)	0.664	105.5 (70; 137.5)	114.5 (83; 134)	0.568
PSI risk class IV–V, *n* (%) ^b^	58 (66)	2,366 (62)	0.411	53 (69)	34 (68)	0.922
SOFA score, median (IQR)	3 (2; 4)	2 (1; 3)	0.087	3 (2; 4)	3 (2; 5)	0.685
Severe CAP, *n* (%)	46 (69)	1,337 (32)	** < 0.001**	43 (67)	35 (56)	0.215
Major criteria	11 (16)	254 (6)	**0.002**	11 (17)	8 (13)	0.502
≥ 3 minor criteria	18 (27)	752 (18)	0.053	16 (25)	12 (19)	0.446
Major and ≥ 3 minor criteria	17 (25)	331 (8)	** < 0.001**	16 (25)	15 (24)	0.916
Bacteremia, *n* (%) ^c^	22 (29)	554 (13)	** < 0.001**	20 (26)	25 (32)	0.377
Pleural effusion, *n* (%)	13 (22)	837 (15)	0.156	13 (22)	15 (20)	0.706
Multilobar involvement, *n* (%)	51 (57)	1,485 (27)	** < 0.001**	44 (56)	44 (56)	> 0.999
ARDS, *n* (%)	21 (26)	271 (5)	** < 0.001**	18 (25)	14 (19)	0.423
Acute renal failure, *n* (%)	31 (44)	1,510 (28)	**0.002**	28 (42)	34 (44)	0.888
Septic shock, *n* (%)	10 (11)	396 (7)	0.150	10 (13)	9 (12)	0.807
Length of hospital stay, median (IQR), days	12 (9; 24)	7 (5; 11)	** < 0.001**	13 (9; 25)	10 (6.5; 15.5)	0.007
ICU admission, *n* (%)	56 (63)	1,040 (19)	** < 0.001**	54 (69)	46 (59)	0.182
ICU mortality, *n* (%) ^d^	5 (9)	116 (11)	0.605	5 (9)	10 (22)	0.082
Length of ICU stay, median (IQR), days ^d^	15.5 (10.5; 29.5)	12 (8; 20)	**0.005**	15.5 (10; 30)	12 (8; 23)	0.074
Mechanical ventilation, *n* (%) ^e^			** < 0.001**			0.198
Non-invasive	3 (5)	183 (4)	0.491	5 (6)	12 (15)	**0.040**
Invasive	24 (40)	360 (7)	** < 0.001**	27 (35)	24 (31)	0.308
In-hospital mortality, *n* (%)	11 (12)	432 (8)	0.112	10 (13)	16 (21)	0.197
3-day mortality, *n* (%)	1 (1)	82 (1)	> 0.999	1 (1)	4 (5)	0.367
30-day mortality, *n* (%)	10 (13)	439 (8)	0.113	9 (12)	15 (19)	0.183
1-year mortality, *n* (%)	13 (23)	600 (11)	**0.004**	12 (18)	16 (21)	0.664

*ARDS*, acute respiratory distress syndrome; *CAP*, community-acquired pneumonia; *IQR*, interquartile range; *ICU*, intensive care unit; *PSI*, Pneumonia Severity Index; *SD*, standard deviation. Percentages calculated with non-missing data only. ^a^Possibly > 1 comorbidity. ^b^Stratified by 30-day mortality risk for CAP: classes I–III (≤ 90 points) had low mortality risk, while classes IV–V (> 90 points) had the highest mortality risk. ^c^Calculated only for patients with blood samples. ^d^Calculated only for patients admitted to intensive care. ^e^Patients who initially received non-invasive ventilation yet subsequently needed intubation were included in the invasive mechanical ventilation group

Bold numbers refers to statistically significant differences

### Microbial aetiology

In the full cohort, microbiologic diagnosis was more frequent in the case group (70% vs. 41%, *p* < 0.001) than in the control group. A higher prevalence of polymicrobial aetiology and *Staphylococcus aureus*, as well as a lower prevalence of *Legionella pneumophila*, was observed in the case group when compared to the control group. After PSM, no differences in microbiologic diagnoses were observed (73% vs. 76%, *p* = 0.714); however, *Staphylococcus aureus* was more frequent in the case group than in the control group (Table 2).

**Table 2:** Microbial aetiology

	CAP (full cohort)			CAP (propensity score matching)		
Variable	(*N* = 5,640)			(*N* = 156)		
	Case (*n* = 89)	Control (*n* = 5,551)	*p* value	Case (*n* = 78)	Control (*n* = 78)	*p* value
Patients with defined aetiology	62 (70)	2,302 (41)	** < 0.001**	57 (73)	59 (76)	0.714
*Streptococcus pneumoniae*	22 (35)	982 (43)	0.259	19 (33)	25 (42)	0.316
*Respiratory virus*	13 (21)	335 (15)	0.159	13 (23)	6 (10)	0.066
Polymicrobial	21 (34)	320 (14)	** < 0.001**	19 (33)	20 (34)	0.949
Atypical	0 (0)	119 (5)	0.073	0 (0)	2 (3)	0.496
*Mycoplasma pneumoniae*	0 (0)	54 (2)	0.401	0 (0)	1 (2)	> 0.999
*Coxiella burnetii*	0 (0)	22 (1)	> 0.999	0 (0)	1 (2)	> 0.999
*Chlamydophila pneumoniae*	0 (0)	41 (2)	0.626	0 (0)	0 (0)	-
*Chlamydophila psittaci*	0 (0)	2 (0.1)	> 0.999	0 (0)	0 (0)	-
*Legionella pneumophila*	0 (0)	151 (7)	**0.031**	0 (0)	0 (0)	-
*Staphylococcus aureus*	5 (8)	61 (3)	**0.028**	5 (9)	0 (0)	**0.026**
*Haemophilus influenzae*	0 (0)	97 (4)	0.180	0 (0)	1 (2)	> 0.999
*Pseudomonas aeruginosa*	1 (2)	87 (4)	0.728	1 (2)	2 (3)	> 0.999
Gram-negative *Enterobacteriaceae*	0 (0)	51 (2)	0.643	0 (0)	1 (2)	> 0.999
*Moraxella catarrhalis*	0 (0)	6 (0.3)	> 0.999	0 (0)	0 (0)	-
Other *Streptococcus species*	0 (0)	6 (0.3)	> 0.999	0 (0)	0 (0)	-
Others	0 (0)	82 (4)	0.275	0 (0)	2 (3)	0.496

*CAP*, community-acquired pneumonia. Results are given as *n* (%). Percentages calculated on non-missing data. Pathogen percentages are related to the number of patients with an aetiologic diagnosis in each group

### Outcomes

In the full cohort, the case group had more ICU admissions; longer length of hospital stay; longer length of ICU stay; more invasive mechanical ventilation; and higher 1-year mortality (Table 1). Cox regression (Table 3) showed that the risks of in-hospital, 3-day, and 30-day mortality did not differ between patients of either the case or control groups. After PSM, the case group was associated with a lower in-hospital mortality (adjusted HR 0.41 [95% CI 0.18 to 0.92]) and a longer length of hospital stay in comparison to the control group (Table 1 and Table 3, respectively).

**Table 3:** Cox regression models evaluating the risk of in-hospital and 30-day mortality in the case group

Variable	HR	95% CI	*p* value
In-hospital mortality			
Crude (full cohort)	0.72	0.39 to 1.31	0.279
Propensity score matching	0.41	0.18 to 0.92	**0.031**
3-day mortality			
Crude (full cohort)	0.76	0.11 to 5.44	0.782
Propensity score matching	0.24	0.03 to 2.19	0.208
30-day mortality			
Crude (full cohort)	1.41	0.75 to 2.63	0.286
Propensity score matching	0.54	0.24 to 1.24	0.149

*HR*, hazard ratio; *CI*, confidence interval

## Discussion

There are 3 main findings of this study. First, ceftaroline was mainly prescribed in cases of severe pneumonia with high suspicion of *S. aureus* infection. Second, in-hospital mortality of the PSM cohort was 13% in the ceftaroline group, and 21% in the control group. Third, after confounding variables were adjusted, the use of ceftaroline was associated with a lower in-hospital mortality rate.

Implementing new antibiotics into clinical practice often implies use of such drugs in the most severe cases, and a simple analysis of its effectiveness could be biased. Ceftaroline has been proposed as a better alternative to ceftriaxone during influenza season when *S. aureus* is more prevalent [17]. Severe infections characterize this population [18, 19], and it explains why ceftaroline was used mainly in critically ill patients in our cohort (67% presented with severe CAP). These patients had a higher prevalence of *S. aureus* and a recent history of pneumococcal vaccine. Ceftaroline is 16 times more active against MSSA (MIC_90_ 0.25 versus 4 mg/L) than ceftriaxone and is active against MRSA as well [11]. Furthermore, available data about critically ill patients suggest that 2 g of ceftriaxone does not reach adequate plasma concentrations [20]. In the case of ceftaroline, pharmacokinetic/pharmacodynamic (PK/PD) target is achieved with a standard dosage, although PK data in critically ill patients is lacking. In addition, an animal model of pneumonia due to MRSA-producing Panton-Valentine leukocidin (PVL) showed that ceftaroline was bactericidal and also significantly reduced PVL concentration in the lung [21]. All of this data could explain why better results were obtained in 3 randomized controlled trials (RCT) [22–24] comparing the clinical efficacy of ceftaroline vs. ceftriaxone. In a meta-analysis of these studies, which included 1916 patients, ceftaroline (600 mg/12 h) was superior to ceftriaxone (1–2 g/24 h) in terms of clinical recovery (OR 1.66; 95% CI 1.34, 2.06) [25]; however, mortality rate in these studies was low (1.5% in each group) [25].

In our study, in-hospital mortality of the PSM cohort was 15%. This is a high mortality for CAP [26, 27], clearly demonstrating the severity of patients included in the analysis. Interestingly, in-hospital mortality was lower in the ceftaroline group (13% vs. 21%, *p* = 0.197). After we adjusted the analysis for confounding variables, in-hospital mortality was significantly lower in the ceftaroline group (adjusted HR 0.41 [95% CI 0.18 to 0.92]). The same trend was observed in results obtained for 30-day and 1-year mortality (adjusted HR 0.54 [95% CI 0.24 to 1.24]); however, the small number of patients may have not allowed for significance to be reached. Longer in-hospital survival in the case group may explain extended hospital stay.

There is a continuous debate about which antibiotic, a macrolide or fluoroquinolone, is the best companion to β-lactams. Both options are included as first-line choices in recent ATS guidelines [1]. Several studies have reported an association between the use of the combination of β-lactams plus macrolide and lower mortality in CAP, when compared to the use of β-lactams in monotherapy [28, 29]. However, Postma et al. [30]. found that β-lactam monotherapy was not inferior to the treatment with combination of β-lactams and macrolides or fluoroquinolone monotherapy with respect to 90-day mortality in patients with non-severe CAP. The main benefits of macrolides include a reduction in pneumolysin, immunomodulation, and activity against atypical pathogens, albeit not against *S. aureus* [31]. An association of macrolide with levofloxacin offers activity against *S. aureus*, but there is little data about its efficacy and some studies have even shown a worse prognosis [28]. In addition, the use of fluoroquinolones is associated with severe adverse effects [32, 33]. The potent activity of ceftaroline against *S. aureus*, the possibility to combine it with a macrolide, and the present article showing good outcomes in patients with severe CAP highlight how such a combination could offer broad-spectrum coverage, potent bactericidal activity, and the potential benefits of macrolides.

We believe that our statistical approach using a PSM is a strength in this study. There are, however, some limitations due to the final total sample size. In this study, we were able to analyze only 78 patients in each group (total *N* = 156). This sample size may result in a large type II error, and conclusions that can be drawn are limited. This aside, though, the rigorous approach to the study underpins our confidence in its findings and their clinical relevance. Another limitation of this study is the analysis of empiric antibiotic therapy, without consideration of dose or duration of the treatment.

Our data showed that ceftaroline was associated with a decreased risk of mortality in hospitalized patients with severe CAP. Ceftaroline is a very active β-lactam against *S. pneumoniae* and *S. aureus*, which can be associated with a macrolide without a loss of beneficial effects. This explains why ceftaroline was recommended by current ATS/IDSA guidelines [1] in the management of CAP. Our experience supports the use of ceftaroline in patients with severe CAP.

## Supplementary Information

Below is the link to the electronic supplementary material.

Supplementary file1 (DOCX 16 KB)
