# Supplementary material for: Impact on in-hospital mortality of ceftaroline versus standard of care in community-acquired pneumonia: a propensity-matched analysis
Source: Eur J Clin Microbiol Infect Dis. 2021 Nov 12;41(2):271–9. doi: 10.1007/s10096-021-04378-0 (PMC8588767; doi:10.1007/s10096-021-04378-0)
Supplement: Supplementary file 1 — Supplementary file1 (DOCX 16 KB) [file 10096_2021_4378_MOESM1_ESM.docx]

**Supplementary material**

**Impact on in-hospital mortality of Ceftaroline versus standard of care in Community-Acquired Pneumonia: A Propensity Matched Analysis**

Catia Cilloniz^1^, Raúl Mendez^2^, Héctor Peroni^3^, Carolina Garcia-Vidal^4^, Verónica Rico^4^, Albert Gabarrus^1^, Rosario Menéndez^2^, Antoni Torres^1^, Alex Soriano^4^

^1^Department of Pneumology, Hospital Clinic of Barcelona; August Pi i Sunyer Biomedical Research Institute - IDIBAPS, University of Barcelona; Biomedical Research Networking Centers in Respiratory Diseases (CIBERES) Barcelona, Spain.

^2^Department of Pneumology, Hospital La Fe de Valencia, Valencia, Spain.

^3^Internal Medicine Department, Respiratory Medicine Unit and Emergency Department, Hospital Italiano de Buenos Aires, Buenos Aires, Argentina.

**Correspondence:** Prof. Alex Soriano (primary) or Dr Catia Cillóniz (alternative)

Department of Infectious Diseases, Hospital Clinic of Barcelona

C/ Villarroel 170, 08036 Barcelona, Spain

Tel: (+34) 93-227-5400 ext. 2887

Email: asoriano@clinic.cat (primary) or catiacilloniz@yahoo.com (alternative)

**Online Table 1. Antimicrobial in full cohort and in the propensity score matching**

| Antimicrobial, n (%) | **Full cohort** | | **Propensity score matching** | |
| --- | --- | --- | --- | --- |
|  | Case  **(n = 89)** | Control  **(n = 5,551)** | Case  **(n = 78)** | Control  **(n = 78)** |
| ß-lactamics | 7 (8) | 511 (9) | 6 (8) | 5 (6) |
| Macrolides | 0 (0) | 31 (0.6) | 0 (0) | 3 (4) |
| Fluoroquinolones | 0 (0) | 892 (16) | 0 (0) | 7 (9) |
| Others | 0 (0) | 19 (0.3) | - | - |
| ß-lactamics + fluroquinolones | 17 (19) | 1133 (20) | 12 (15) | 23 (29) |
| ß-lactamics + macrolides | 41 (46) | 2333 (42) | 40 (51) | 27 (35) |
| Other combinations | 24 (27) | 632 (11) | 20 (26) | 13 (17) |
